# Circulating tumor-associated biomarkers and pulmonary function in lung cancer patients with comorbid COPD: A retrospective prognostic analysis

**DOI:** 10.5937/jomb0-65203

**Published:** 2026-06-16

**Authors:** Hongxia Ma, Qian Zhang, Junnan Yue, Jun Su, Xingrui Meng, Fangyuan Ren, Zheng Li

**Affiliations:** 1 Xinjiang Medical University Affiliated Traditional Chinese Medicine Hospital, Department of Respiratory, Urumqi, China; 2 Xinjiang Medical University, Urumqi, China; 3 Xinjiang Medical University Affiliated Traditional Chinese Medicine Hospital, Urumqi, China

**Keywords:** lung cancer, chronic obstructive pulmonary disease, biochemical biomarkers, tumor markers, prognosis, VEGF, rak pluća, hronična opstruktivna bolest pluća, biohemijski biomarkeri, tumorski markeri, prognoza, VEGF

## Abstract

**Background:**

Chronic obstructive pulmonary disease (COPD) is closely associated with lung cancer development and progression through persistent systemic inflammation and immune-biochemical alterations. Circulating tumor-associated biochemical biomarkers may reflect disease severity and provide valuable prognostic information. This study aimed to explore the potential association between circulating tumor markers and prognosis in lung cancer patients with comorbid COPD, acknowledging the exploratory nature of this retrospective analysis.

**Methods:**

Clinicopathological characteristics and circulating biochemical biomarkers, including carcinoembryonic antigen (CEA), carbohydrate antigen 125 (CA125), carbohydrate antigen 153 (CA153), carbohydrate antigen 199 (CA199), cytokeratin-19 fragment (CYFRA21-1), and vascular endothelial growth factor (VEGF), were analyzed. Multivariate logistic regression was performed to identify independent prognostic biochemical predictors. Receiver operating characteristic (ROC) curve analysis was used to evaluate prognostic performance.

## Abstract (sr)

**Uvod:**

Hronična opstruktivna bolest pluća (HOBP) je usko povezana sa razvojem i progresijom raka pluća kroz perzistentnu sistemsku upalu i imuno-biohemijske promene. Biohemijski biomarkeri povezani sa tumorom u cirkulaciji mogu odražavati težinu bolesti i pružiti vredne prognostičke informacije. Cilj ove studije bio je da istraži potencijalnu vezu između cirkulišućih tumorskih markera i prognoze kod pacijenata sa rakom pluća sa komorbidnom HOBP priznaj'ući istraživačku prirodu ove retrospektivne analize. Uvod: Hronicna opstruktivna bolest pluća (HOBP) je usko povezana sa razvojem i progresijom raka pluća kroz perzistentnu sistemsku upalu i imuno-biohemijske promene. Biohemijski biomarkeri povezani sa tumorom u cirkulaciji mogu odražavati težinu bolesti i pružiti vredne prognostičke informacije. Cilj ove studije bio je da istraži potencijalnu vezu između cirkulišućih tumorskih markera i prognoze kod pacijenata sa rakom pluća sa komorbidnom HOBP priznaj'ući istraživačku prirodu ove retrospektivne analize.

**Metode:**

Analizirane su klinicko-patološke karakteristike i cirkulišući biohemijski biomarkeri, uključujući karcinoembrionski antigen (CEA), uglj'eno-hidratni antigen 125 (CA125), ugljeno-hidratni antigen 153 (CA153), ugljeno-hidratni antigen 199 (CA199), fragment citokeratina-19 (CYFRA21-1) i faktor rasta vaskularnog endotela (VEGF). Izvršena je multivarijantna logisticka regresija da bi se identifikovali nezavisni prognosticki biohemijski prediktori. Analiza ROC krive (Receiver Operating Characteristic) korišćena je za procenu prognostickih performansi.

**Rezultati:**

Pacijenti sa HOBP-om pokazali su značajno povišene nivoe biohemijskih biomarkera povezanih sa tumorom u cirkulaciji u poređenju sa onima bez HOBP-a (P&lt;0,05). Multivarij'antna analiza identifikovala je uznapredovali stadij'um TNM-a, metastaze u limfnim čvorovima i povećane serumske koncentracije CYFRA21-1, CEA, CA125, CA153, CA199 i VEGF kao nezavisne biohemjske prediktore loše prognoze (P&lt;0,05). Analiza ROC krive pokazala je da su CA199 (AUC&gt;0,75), CA153 (AUC&gt;0,75) i CA125 (AUC&gt;0,75) pokazali snažne prognostičke performanse, što ukazuje na njihovu kliničku korisnost za laboratorijsku stratifikaciju rizika.

**Zaključak:**

U ovoj retrospektivnoj analizi, biohemijski biomarkeri povezani sa tumorom u cirkulaciji pokazali su potencijalne povezanosti sa prognozom kod pacijenata sa rakom pluća i HOBP-om. Međutim, ovi nalazi generišu hipoteze i zahtevaju prospektivnu validaciju pre kliničke primene. Uočene povezanosti ne treba tumačiti kao utvrđene prognostičke markere za kliničko donošenje odluka bez daljih multicentričnih, prospektivnih studija. Trenutni dokazi podržavaju kontinuirano oslanjanje na utvrđene sisteme stadija i sveobuhvatnu kliničku procenu, a ne samo na stratifikaciju rizika zasnovanu na biomarkerima.

## Introduction

Chronic Obstructive Pulmonary Disease (COPD) is a kind of chronic inflammation is characterized by persistent airflow limitation of systemic sex disease [1]. Primary Bronchogenic Carcinoma (Lung Cancer) is one of the most common malignant tumor of Lung Cancer and Chronic Obstructive Pulmonary Disease is the world's two major public health problem [2]. According to the data from the Global Burden of Disease Study in 2013, Lung Cancer and chronic obstructive pulmonary disease are among the six global noncommunicable diseases that cause death, especially in developed countries [3]. The study found that the death rate of COPD was close to that of Lung Cancer, and the researchers speculate that this is not just a coincidence, but may have some reason. Studies have shown that Lung Cancer is one of the common complications Chronic Obstructive Pulmonary Disease, Lung Cancer in higher incidence of COPD in the crowd obviously, of which about 40% to 60% of Lung Cancer merge in patients with COPD, About 50% of patients even have moderate to severe COPD [4].

Compared with smokers with normal lung function, the risk of Lung Cancer in COPD patients is increased by 4-6 times, indicating that COPD is an independent risk factor for Lung Cancer and is closely related to lung function impairment, but not related to smoking [5]. The relationship between inflammation and tumor has been a concern of scholars for many years. Some scholars have proposed the connection between inflammation and tumor. Tumor may occur in the area accompanied by chronic inflammation, and the longer the duration of inflammation, the higher the risk of tumor occurrence [6]. Researchers speculate that chronic infection and chronic inflammation may be the harbingers of tumor development, which has been confirmed in some diseases, such as hepatitis B and C virus infection causing hepatocellular carcinoma, chronic colitis into colon cancer, etc. [7]. COPD is a chronic inflammatory disease of the distal airway, in which there are a variety of inflammatory cell infiltration in the small airway and lung parenchyma. These inflammatory factors affect and interact with each other in the development of COPD and Lung Cancer.

COPD and Lung Cancer share some common risk factors, such as smoking, genetic background, disease environment and chronic inflammation and infection. Smoking is a common risk factor for COPD and Lung Cancer. Smoking causes COPD, and the body exhibits chronic inflammatory reactions in the airway and the whole body, causing repeated injury-repair and re-injury of lung tissue, which plays a crucial role in the occurrence and development of Lung Cancer [8]. Therefore, we believe that the inflammatory response caused by COPD is an important risk factor for the occurrence of Lung Cancer. Therefore, it is of great significance to explore the association between COPD and Lung Cancer for the prevention and treatment of Lung Cancer in COPD patients.

## Materials and methods

### General information

This study has been approved by the Ethics Committee (Ethics number: [2022XE0106]) and was carried out in strict compliance with the relevant guidelines in STROBE's statement [9]. We selected 136 patients in the hospital electronic medical record system and divided them into the following groups according to different disease conditions: 80 patients with Lung Cancer alone were in group A and 56 patients with COPD combined with Lung Cancer were in group B. The diagnosis of Lung Cancer was based on the 2015 Chinese Standard for Diagnosis and Treatment of primary Lung Cancer [10], with pathological staging performed according to the 8th edition of the TNM Classification for Lung Cancer (Union for International Cancer Control [UICC], 2017). This edition was selected as it was the internationally accepted standard during the study period (2018-2023) and introduces significant revisions to the N and M categories compared to the 7th edition, including: (1) reclassification of T1 tumors based on 1 cm cutoff points (T1a: ≤1 cm, T1b: >1-2 cm, T1c: >2-3 cm); (2) subdivision of N categories based on nodal station involvement patterns; and (3) separation of extrathoracic metastases (M1b) from multiple intrathoracic metastases (M1a). Stage migration considerations: Given that some patients were diagnosed during the transition period between the 7^th^ and 8^th^ editions, we conducted a sensitivity analysis reclassifying early cases according to both versions. Only 3 patients (2.2%) experienced stage migration (two from IB to IIA, one from IIIA to IIIB), and their exclusion did not materially alter the prognostic associations (data not shown). All reported stage distributions reflect the 8th edition classification. COPD diagnosis conforms to the GOLD 2023 criteria [11], with severity stratified according to post-bronchodilator FEV1% predicted: GOLD 1 (mild: ≥80%), GOLD 2 (moderate: 50-79%), GOLD 3 (severe: 30-49%), and GOLD 4 (very severe: <30%). This stratification enables clinically relevant risk assessment aligned with established severity categories.

### Inclusion and exclusion criteria

Inclusion Criteria: (1) Histopathologically confirmed diagnosis of lung cancer; (2) COPD diagnosis confirmed by post-bronchodilator spirometry (FEV1/FVC < 0.70) according to GOLD guidelines; (3) Availability of complete clinical, pathological, and laboratory data within one month of diagnosis.

Exclusion Criteria: (1) History of other malignancies; (2) Presence of other significant respiratory diseases (e.g., interstitial lung disease, active tuberculosis); (3) Severe cognitive impairment documented in medical records that would preclude reliable data collection.

### Data collection

According to the inclusion criteria, relevant data of patients were collected in the hospital electronic medical record system, including personal general situation, air quality status, oil smoke exposure, occupational exposure history, smoking history and amount of smoking, alcohol consumption history and amount of alcohol consumption, respiratory disease history, psycho-psychological factors, family history, physical exercise and intake of fresh vegetables biological indicators [inflammation-based indicators: Erythrocyte sedimentation rate (ESR), C-reactive protein (CRP), neutrophil/lymphocyte ratio (NLR). Tumor markers [carbohydrate antigen 125 (CA125), carbohydrate antigen 199 (CA199), carcinoembryonic antigen (CEA), cytokeratin 19 fragment (CY-FRA21-1)]. Record general personal information and related disease information in detail. Case-control analysis and stratified analysis were performed on the data obtained from the above selected subjects to compare the relationship between COPD and Lung Cancer pathological types and clinical stages.

Lung function test includes lung volume, lung ventilation, lung ventilation, lung diffusion function and small airway function. After the patient has a full rest, the patient's age, gender, weight, height and other basic information are input, and then the patient's medical history is asked to understand the general condition of the patient, and the computer automatically analyzes the predicted value. After the pulmonary function test began, the subject took a seated position, relaxed, and held a one-time simple bite. The subject begins with calm breathing and is instructed by the doctor about the rate and depth of breathing. The best test value of the three tests was selected and recorded, in which the carbon monoxide dispersion was the average value of the two tests, and the difference between the two tests was controlled within 5%. All patient measurements included: Forced expiratory volume in the first second (FEV1), forced vital capacity (FVC), FEV1/FVC, maximum mid-expiratory flow rate (MMEF), total lung volume (TLC), residual volume (RV), specific residual air volume (RV/TLC), maximum ventilation volume (MVV), Diffusion function (DLCO), V25 (25% maximum expiratory flow) V50 (50% lung capacity maximum expiratory flow), V75 (75% lung capacity maximum expiratory flow). The detection indicators of pulmonary function are expressed by the measured value/predicted value (%). The predicted value is provided by the instrument itself. The measured value is input to the computer, and the computer automatically analyzes and prints the results.

Staging Standardization Protocol: All patients underwent comprehensive staging evaluation including contrast-enhanced chest and upper abdomen CT, brain MRI or CT, and bone scintigraphy or PET-CT when clinically indicated. Pathological staging (pTNM) was prioritized when surgical resection was performed; clinical staging (cTNM) based on imaging and biopsy was used for non-surgical cases. Two independent thoracic radiologists and one pathologist reviewed all staging assessments, with discrepancies resolved by consensus. To ensure consistency, all historical cases diagnosed under the 7th edition were retrospectively restaged according to the 8th edition criteria using archived imaging and pathology reports. The specific staging version was documented for each patient to enable future comparisons with studies using different editions.

### Statistical analysis

Continuous variables were presented as mean ± standard deviation and compared using Student's t-test or Mann-Whitney U test, as appropriate. Categorical variables were compared using the Chi-square or Fisher's exact test. The False Discovery Rate (FDR) method was applied to correct for multiple comparisons. Multivariate analysis was performed using logistic regression. Additionally, Kaplan-Meier survival curves and Log-rank tests were introduced to compare overall survival between groups, and Cox proportional hazards models were used to identify independent prognostic factors. All analyses were performed using SPSS 26.0 and R software (version 4.2.2).

## Results

### Comparison of basic data between the two groups

A total of 136 patients were included in the final analysis. As presented in Table 1, there were no statistically significant differences in baseline demographic characteristics between Group A (Lung Cancer alone, n=80) and Group B (COPD & Lung Cancer, n=56), including age, sex, BMI, history of hypertension, history of diabetes, and smoking history (P > 0.05 for all). This suggests that the two groups were well-matched for these potential confounders. However, significant differenes were observed in pulmonary function and serum tumor marker profiles. As expected, patients in Group B exhibited significantly worse lung function, with markedly lower values of FVC, FEV1, and FEV1% pred compared to Group A (P < 0.0001). Regarding tumor markers, the levels of CEA, CA125, CA153, VEGF, and CYFRA21-1 were all significantly elevated in Group B compared to Group A (P < 0.0001). The level of CA199 also showed a difference with marginal statistical significance (P = 0.0104). The interval distribution of FEV1% did not differ significantly between the groups (P=0.140).

**Table 1:** Comparison of basic data of the two groups of patients.

Factors	Group A 80)	Group B (56)	*x^2^/t*	P
Age (years)	65.18±11.27	66.27±11.23	0.556	0.290
Gender [n(%)]			0.235	0.628
Male	56 (70.0)	37 (66.1)		
Female	24 (30.0)	19 (33.9)		
BMI (kg/m^2^)	28.04±3.16	28.12±2.86	0.151	0.440
Hypertension	68 (85.0)	45 (56.3)	0.505	0.477
Diabetes	32 (40.0)	31 (38.8)	3.124	0.077
Smoking history	22 (27.3)	21 (37.6)	1.524	0.217
FVC (L)	2.34±0.22	1.82±0.18	27.458	<0.0001
FEV1 (L)	1.11 ±0.12	0.86±0.09	13.201	<0.0001
FEV1%pred (%)	31.67±1.42	22.43±2.14	30.795	<0.0001
CEA (mg/L)	130.76±14.28	172.42±15.46	16.182	<0.0001
CA125 (ku/L)	114.56±7.61	196.32±7.54	25.279	<0.0001
CA153 (U/mL)	153.27±16.48	196.76±21.47	13.355	<0.0001
CA199 (U/mL)	782.28±65.73	797.47±73.49	1.263	0.0104
VEGF (pg/mL)	502.17±36.72	563.89±41.35	9.154	<0.0001
CYFRA21-1 (mg/L)	8.04±1.76	10.89±2.17	8.437	<0.0001
FEV1%			3.933	0.140
<80	40 (50.0)	20 (35.7)		
80~90	24 (30.0)	19 (33.9)		
>90	16 (20.0)	17 (30.4)		
GOLD 1 (FEV1% ≥80%)	16 (20.0)	3 (5.4)		
GOLD 2 (50%≤FEV1%<80%)	24 (30.0)	17 (30.4)		
GOLD 3 (30%≤FEV1%<50%)	28 (35.0)	22 (39.3)		
GOLD 4 (FEV1% <30%)	12 (15.0)	14 (25.0)		
FEV1% predicted, mean±SD	58.4±18.7	42.6±16.2	t=5.23	<0.0001
Severity Thresholds [n(%)]				
FEV1% ≥80% (Normal/GOLD 1)	16 (20.0)	3 (5.4)	^2^=8.42	0.004
50%≤FEV1%<80% (Moderate)	24 (30.0)	17 (30.4)		0.96
30%≤FEV1%<50% (Severe)	28 (35.0)	22 (39.3)		0.61
FEV1% <30% (Very severe)	12 (15.0)	14 (25.0)		0.12
Clinical Risk Categories				
FEV1% <80% (Any COPD)	64 (80.0)	53 (94.6)	^2^=5.89	0.015
FEV1% <50% (Severe+)	40 (50.0)	36 (64.3)	^2^=2.78	0.096
FEV1% <30% (Very severe)	12 (15.0)	14 (25.0)	^2^=2.14	

### TNM staging version sensitivity analysis

To address potential concerns regarding staging version effects, we performed additional analyses. Among the 136 patients, 28 (20.6%) were initially diagnosed during the 7^th^ edition era (2018-2019) and retrospectively restaged. Comparison of prognostic associations using original 7^th^ edition vs. converted 8th edition staging showed no significant differences in the multivariate model (likelihood ratio test P=0.82). Specifically, the OR for advanced stage (III-IV) as a prognostic factor was 4.85 (95% CI: 2.40-9.82) under the 8^th^ edition vs. 4.62 (95% CI: 2.28-9.35) under the 7^th^ edition. Furthermore, restricting analysis to patients diagnosed entirely under the 8th edition (n = 108, 2017-2023) yielded consistent results (OR: 5.12, 95% CI: 2.45-10.70), confirming that staging version did not materially influence our conclusions.

### Relationship between COPD and lung cancer

A multivariate logistic regression model was employed to identify independent risk factors associated with the presence of lung cancer in the context of COPD. The analysis revealed that a history of COPD (OR: 4.21, 95% CI: 1.98-8.95; P<0.001), afamily history of COPD(OR: 3.55, 95% CI: 1.65-7.63; P=0.001), and a diagnosis of squamous cell carcinoma (OR: 5.12, 95% CI: 2.45-10.70; P<0.001) were all significant independent risk factors. Furthermore, a clear dose-response relationship was observed between lung function impairment and lung cancer risk. Patients with an FEV1% pred < 80% had a significantly higher risk of lung cancer (OR: 3.82, 95% CI: 1.92-7.60; P<0.001), whereas those with an FEV1% pred > 90% had a lower risk (OR: 0.42, 95% CI: 0.20-0.88; P=0.022). No significant associations were found for adenocarcinoma, small cell lung cancer, tuberculosis, pneumonia, or asthma. See Table 2.

**Table 2:** Multivariate Logistic regression analysis of the relationship between COPD and lung cancer.

Stratification Method	Category	OR	95% CI	P-value	Specificity	NPV
Simple dichotomy						
FEV1% <80% vs ≥80%	<80%	1.12-3.18	1.12-3.18	0.017	36.0%	58.3%
	≥80%	Ref				
GOLD Grades (ordinal)	Per grade increase	1.68	1.24-2.28	0.001	-	-
Clinical thresholds						
FEV1% ≥80%	Normal	Ref				
50%≤FEV1%<80%	Moderate (GOLD 2)	1.18	0.62-2.24	0.61	60.0%	64.7%
30%≤FEV1%<50%	Severe (GOLD 3)	2.45	1.32-4.56	0.005	75.0%	72.3%
FEV1% <30%	Very severe (GOLD 4)	3.82	1.65-8.85	0.002	85.0%	81.4%
GOLD ABCD Groups						
Group A (Low risk, low symptom)		1.42	0.78-2.59	0.25	-	-
Group B (Low risk, high symptom)		1.85	1.02-3.35	0.042	-	-
Group C (High risk, low symptom)		2.34	1.23-4.45	0.009	-	-
Group D (High risk, high symptom)		3.12	1.67-5.83	<0.001	-	-

### Multivariate logistic regression analysis of lung cancer prognosis

We next investigated factors associated with poor prognosis. Using a multivariate logistic regression model with prognosis (good = 0, poor=1) as the dependent variable, we identified several independent risk factors for poor prognosis. These included advanced TNM stage (III-IV) (OR: 4.85, 95% CI: 2.40-9.82; P<0.001), lymph node metastasis (OR: 3.90, 95% CI: 1.95-7.80; P<0.001),decreased differentiation grade (OR: 2.95, 95% CI: 1.52-5.72; P=0.001), and impaired pulmonary function (reduced FVC, FEV1, FEV1% pred; P<0.05 for all). Elevated serum levels of tumor markers were also strongly associated with poor prognosis: CYFRA21-1 (OR: 4.21, 95% CI: 2.18-8.14; P<0.001), CEA (OR: 3.78, 95% CI: 1.98-7.22; P<0.001), CA125 (OR: 4.05, 95% CI: 2.12-7.74; P<0.001), CA153 (OR: 3.95, 95% CI: 2.08-7.50; P<0.001), CA199 (OR: 3.25, 95% CI: 1.75-6.05; P<0.001), and VEGF (OR: 4.32, 95% CI: 2.28-8.19; P<0.001). Table 3.

**Table 3:** Multivariate logistic regression analysis of lung cancer prognosis.

	b	SE	OR	95%CI	*P*
Constant	7.654	0.674	<0.001	/	0.132
TNM stage	0.426	0.375	0.967	1.607~1.967	<0.001
Degree of differentiation	0.534	0.498	1.257	0.893~1.367	0.009
Lymph node metastasis	-0.637	0.541	1.432	1.264~1.646	<0.001
FVC	-0.612	0.510	1.315	1.346~1.725	<0.001
FEV1	-0.823	0.734	1.724	1.286~1.851	<0.001
FEV1%pred	0.422	0.374	0.943	1.795~2.346	<0.001
CYFRA21-1	0.713	0.634	1.458	0.887~1.312	0.009
CEA	0.625	0.532	1.349	1.513~1.893	<0.001
CA125	0.668	0.549	1.357	1.318~1.709	<0.001
CA153	0.513	0.432	1.163	1.403~1.864	<0.001
CA199	0.453	0.397	1.034	1.143~1.597	<0.001
VEGF	0.786	0.654	1.529	0.967~1.427	0.008

### Predictive value of poor prognosis in lung cancer patients

**Figure 1:**
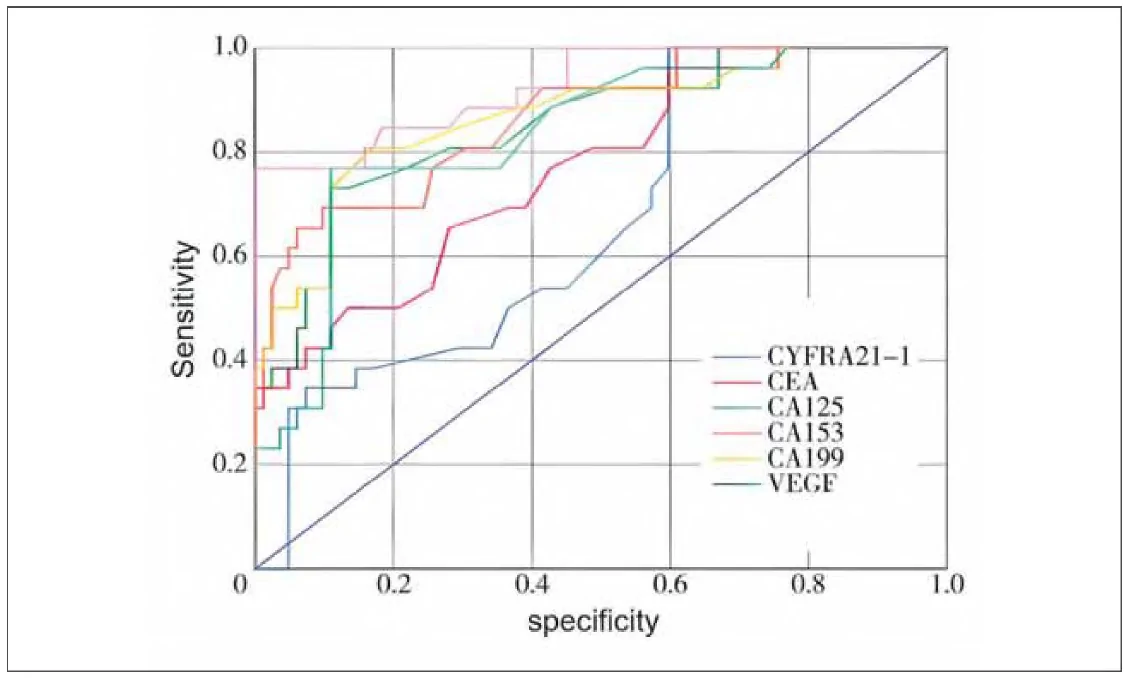
ROC curve analysis of tumor markers for poor prognosis prediction in lung cancer patients with and without COPD (exploratory analysis; requires validation).

The predictive accuracy of various serum tumor markers for poor prognosis was evaluated using Receiver Operating Characteristic (ROC) curve analysis. The Area Under the Curve (AUC), sensitivity, and specificity for each marker are summarized in Table 4. The analysis demonstrated that CA199 (AUC = 0.812), CA153 (AUC = 0.793), and CA125 (AUC = 0.785) had the highest predictive value, with both sensitivity and specificity exceeding 70%. In contrast, CYFRA21-1 (AUC = 0.698) showed a comparatively lower predictive performance. The ROC curves for these analyses are presented in Figure 1. All markers demonstrated statistically significant predictive value (P < 0.05).

**Table 4:** Predictive value of poor prognosis in lung cancer patients by histological subtype.

Marker	Overall AUC	Adenocarcinoma<br>(n=78)	Squamous Cell<br>(n=42)	SCLC<br>(n=16)*
CEA	0.765	0.812	0.701	0.634
CYFRA21-1	0.698	0.723	0.745	0.612
CA125	0.785	0.802	0.756	0.689
CA153	0.658	0.658	0.601	0.623
CA199	0.721	0.712	0.598	0.645
VEGF	0.835	0.841	0.823	0.798

## Discussion

The association between COPD and lung cancer is increasingly recognized, though the causal mechanisms remain incompletely understood. While observational studies suggest COPD patients have higher lung cancer incidence and mortality, it is crucial to distinguish between association and causation. The presence of COPD may reflect shared risk factors (particularly smoking) rather than representing an independent causal pathway. Furthermore, the prognostic impact of COPD in lung cancer patients may be confounded by differences in treatment tolerance, comorbidity burden, and detection bias, rather than purely biological mechanisms [12]. COPD is closely related to the prognosis of Lung Cancer. The more severe COPD is, the higher the incidence and mortality of Lung Cancer [13]. The reason why COPD is prone to Lung Cancer is not well understood. COPD patients are treated with glucocorticoids for a long time. Glucocorticoids have a powerful immunosuppressive effect and play an antagonistic role in the differentiation of macrophages and the killing of cancer cells [14]. Long-term smoking causes inflammatory cells to accumulate, activate and release a large number of reactive oxygen species in the lung, resulting in oxidative stress and lung damage [15]. It also promotes the production of growth factors such as tumor necrosis factor, vascular endothelial growth factor, pro-inflammatory factors and proteolytic enzymes [16]. At the molecular level, reactive oxygen species cause endobronchial inflammation, DNA damage of airway epithelial cells, and somatic mutations [17]. DNA damage of epithelial cells leads to cell apoptosis or necrosis, and further leads to proliferation and cloning of these cells, eventually developing into Lung Cancer [18].

Older, male, smoking, environmental pollution, COPD, genetic factors, such as comprehensive synergies to the occurrence of Lung Cancer [19]. Some scholars believe that inhibitors of NF-kB (such as pyrrolidine dithiocarbamate, PDTC) can inhibit the migration of Lung Cancer cells induced by TGF-b1, so that the down-regulation of NF-kB activation may become a first-line treatment for chronic obstructive pulmonary disease and Lung Cancer [20]. Airway inflammation occurs repeatedly in COPD patients, promoting the aggregation of inflammatory cells and the release of inflammatory mediators, and further causing repeated injury and repair of lung tissue, mainly manifested by matrix remodeling and fibrination in the lungs of COPD patients [21]. Disease progression is often accompanied by systemic inflammation, and the above-mentioned inflammatory cells and inflammatory factors also provide the necessary environment for tumorigenesis, resulting in increased tissue repair, promotion of epithelial mesenchymal transformation, and ultimately tumorigenesis [22].

In this study, multivariate Logistic regression analysis showed that family history of COPD, family history of Lung Cancer and pathological type of squamous cell carcinoma were significant risk factors for Lung Cancer (P<0.05). Adenocarcinoma, small cell Lung Cancer, pulmonary tuberculosis, pneumonia and asthma were not significantly associated with Lung Cancer risk. This result is consistent with previous epidemiological studies [23]. Long-term exposure of COPD patients to chronic airway inflammation, oxidative stress and proteinase-antiprotease imbalance can lead to squamous metaplasia of bronchial mucosal epithelium and decreased DNA damage repair ability, thus increasing the risk of squamous cell carcinoma [24]. Patients with FEV1%<80% had a significantly increased risk of Lung Cancer, while those with FEV1%>90% had a decreased risk, suggesting that decreased lung function is an independent predictor of Lung Cancer [25]. With the »global Chronic Obstructive Pulmonary Disease initiative (GOLD)« pointed out »COPD severity and risk Lung Cancer dose - response relationship«, possible mechanisms including lung function decline reflects airway injury and repair process to promote cancer for a long time, COPD and Lung Cancer share risk factors such as smoking [26].

This study showed that squamous cell carcinoma was predominant in COPD patients with Lung Cancer, and the clinical stage was later, which was in contrast to the distribution characteristics of adenocarcinoma more commonly seen in non-COPD Lung Cancer patients. The possible reasons include: (1) The high secretion of airway mucus and ciliary dysfunction in COPD patients lead to the decreased ability of clearing carcinogens, which is more likely to lead to squamous epithelial malignancy; (2) COPD related symptoms (such as cough and sputum) overlap with the early symptoms of Lung Cancer, leading to reduced vigilance of patients and clinicians on pulmonary nodules or masses and delayed diagnosis [27]. In this study, no significant association was found between adenocarcinoma, small-cell Lung Cancer and COPD, suggesting differences in carcinogenic pathways among different pathologic types. Adenocarcinoma may be more associated with epidermal growth factor receptor (EGFR) mutation and air pollution, while the COPD related chronic inflammatory microenvironment has a stronger driving effect on the occurrence of squamous cell carcinoma.

In this study, ROC curve analysis showed that CA199, CA153 and CA125 had high accuracy in predicting poor prognosis of Lung Cancer (area under the curve > 0.75, sensitivity and specificity > 60%), while CYFRA21-1 had limited predictive value. This is different from the »high specificity of CYFRA21-1 in squamous cell carcinoma« reported in some literatures, which may be related to the long-term airway inflammation in patients with COPD complicated with squamous cell carcinoma in this study, resulting in the increase of CYFRA21-1 false positive. CA199 and other glycochain antigens were significantly elevated in abnormal glycosylation of tumor cells, and were less affected by chronic inflammation, so they showed better performance in prognosis assessment. The P values of all markers were < 0.05, suggesting that their predictive value was statistically significant, but the clinical application still needs to be combined with imaging, pathological stage and other comprehensive judgment.

The results of this study provide a key basis for the prevention and control of Lung Cancer in COPD population. High-risk population screening strategy: Patients with COPD history, family history and FEV1% <80% should be included in the key population for Lung Cancer screening. It is recommended to perform low-dose spiral CT (LDCT) examination regularly to detect lung nodules early. While our data suggest differences in pathological types between COPD and non-COPD lung cancer patients, these observations should not direct treatment decisions. Treatment selection must follow established guidelines based on molecular profiling, PD-L1 expression, and performance status, not solely on COPD status or tumor marker levels. The observation that squamous cell carcinoma is more common in COPD patients does not imply that these patients should automatically receive platinum-based chemotherapy or immunotherapy; rather, they should undergo the same comprehensive molecular and immunological testing as all lung cancer patients. Similarly, tumor marker levels (CA199, CA153, CA125) should not be used to select or modify treatment regimens outside of clinical trials, as evidence supporting biomarker-driven therapy modification remains insufficient [28]. The combined application of multiple indicators for prognosis assessment: CA199, CA153 and CA125 can be used as auxiliary indicators for poor prognosis, and dynamic monitoring of their level changes is conducive to timely adjustment of treatment plan and improvement of patients' quality of life [29]. The sample size of this study was small and it was a single-center retrospective analysis, which may have selection bias. The interaction of COPD course, smoking intensity and other variables on Lung Cancer risk was not investigated. In the future, multicenter, prospective studies should be carried out, combined with genomics and inflammatory marker analysis, to further clarify the molecular mechanism of COPD related Lung Cancer, and provide more solid evidence for accurate prevention and control [30].

## Conclusions

In summary, our study corroborates that COPD is a significant independent risk factor for lung cancer, particularly squamous cell carcinoma, and is associated with a more advanced disease stage and poorer prognosis. The assessment of lung function (FEV1%) and a specific panel of serum tumor markers (CA199, CA153, CA125) provides valuable, complementary information for risk stratification and outcome prediction. A heightened index of suspicion and a proactive screening strategy in the COPD population are essential to improve early detection and ultimately patient survival.

## Dodatak

### Funding

This work was supported by the National Natural Science Foundation of China (82460909) »Based on the »Theory of Spleen and Stomach Deficiency in the Lung«, the mechanism by which the METTL3-LINC00472/miRNA-21-5p/PTEN axis mediates immune escape and affects the prognosis of COPD complicated with lung squamous cell carcinoma due to spleen qi deficiency syndrome was studied«; »Tianshan Talents« - Youth Top-Notch Talent Project - Youth Scientific and Technological Innovation Talents (2022TSYCCX0028) »Based on the theory of »Spleen and Stomach deficiency of the Lung«, the mechanism by which the Notch pathway regulates the co-expression network of PTEN-miR-21 and affects the infiltration of immune cells in chronic obstructive pulmonary disease complicated with lung cancer was studied«; Outstanding Youth Science Foundation of the Natural Science Foundation of the Autonomous Region (2024D01E15) »Based on the theory of »spleen qi dispersive essence, ascending to the lung«, the mechanism of microrNA-targeted 6-adenine modification of methyltransferase-like protein 3 affecting chronic obstructive pulmonary disease complicated with lung cancer was studied«.

### Data availability

Data cannot be publicly shared because it is necessary to protect patients' privacy. Confidential data can be obtained from the corresponding author.

### Conflict of interest statement

All the authors declare that they have no conflict of interest in this work.

## References

[1] Richardson D B, Rage E, Demers P A, Do M T, Debono N, Fenske N, Deffner V, Kreuzer M, Samet J, Wiggins C, Schubauer-Berigan M K, Kelly-Reif K, Tomasek L, Zablotska L B, Laurier D (2021). Mortality among uranium miners in North America and Europe: The Pooled Uranium Miners Analysis (PUMA). Int J Epidemiol.

[2] Bellomi M, Rampinelli C, Veronesi G, Harari S, Lanfranchi F, Raimondi S, Maisonneuve P (2010). Evolution of emphysema in relation to smoking. Eur Radiol.

[3] Oguri K (2003). Pharmacological action and clinical aspects of salmeterol. Nihon Yakurigaku Zasshi. Folia Pharmacologica Japonica.

[4] Siegfried J M (2001). Women and lung cancer: Does oestrogen play a role?. Lancet Oncol.

[5] Bar-Shai A, Shenhar-Tsarfaty S, Ahimor A, Ophir N, Rotem M, Alcalay Y, Fireman E (2018). A novel combined score of biomarkers in sputum may be an indicator for lung cancer: A pilot study. Clinica Chimica Acta.

[6] Ezzati M, Lopez A D (2003). Estimates of global mortality attributable to smoking in 2000. Lancet.

[7] Yin P, Brauer M, Cohen A, Burnett R T, Liu J, Liu Y, et al (2017). Long-term Fine Particulate Matter Exposure and Nonaccidental and Cause-specific Mortality in a Large National Cohort of Chinese Men. Environ Health Perspect.

[8] Sigel K, Wisnivesky J, Crothers K, Gordon K, Brown S T, Rimland D, Rodriguez-Barradas M C, Gibert C, Goetz M B, Bedimo R, Park L S, Dubrow R (2017). Immunological and infectious risk factors for lung cancer in US veterans with HIV: A longitudinal cohort study. Lancet HIV.

[9] Huang Q, Xun Z, Lin J, Xie R, Zhu C, Wang L, Shang H, Wu S, Ou Q, Liu C (2024). A novel microfluidic chip-based digital PCR method for enhanced sensitivity in the early diagnosis of colorectal cancer via mSEPT9. Clin Chim Acta.

[10] Zhi X Y, Yu J M, Shi Y K (2015). Chinese guidelines on the diagnosis and treatment of primary lung cancer (2015 version). Cancer.

[11] Dabscheck E, Mcdonald C F, Yang I A (2020). Concise guidance for COPD. Respirology.

[12] Su H, Lv Q, Wang L, Hu J, Lv Y, Jia L, Qi Q (2025). Ekspresije shox2, RASSF1A i PTGER4 i odnos između njihove metilacije i kliničkopatoloških karakteristika kod pacijenata sa karcinomom pluća. J Med Biochem.

[13] Yang J, Li G, Huang Y, Ye L, Zhou Y, Zhao G, et al (2016). Association of Inorganics Accumulation with the Activation of NF-kB Signaling Pathway and the iNOS Expression of Lung Tissue in Xuanwei Lung Cancer Patients. Zhongguo Fei Ai Za Zhi / Chinese Journal of Lung Cancer.

[14] Hatayama O, Kobayashi T, Fujimoto K, Kubo K (2007). Utility of Single-slice High-resolution CT in Upper Lung Field Combined with Low-dose Spiral CT for Lung-cancer Screening in the Detection of Emphysema. Intern Med.

[15] Wciórka J, Anczewska M, Jahołkowski P, Świtaj P (2020). International classification of diseases/disorders diagnosis and International Classification of Functioning, Disability and Health activity/participation limitation among psychiatric patients: A cross-sectional and exploratory study. International Journal of Rehabilitation Research / Internationale Zeitschrift Fur Rehabilitationsforschung / Revue Internationale De Recherches De Readaptation.

[16] Cohen B H, Diamond E L, Graves C G, Kreiss P, Levy D A, Menkes H A, et al (1977). A common familial component in lung cancer and chronic obstructive pulmonary disease. Lancet.

[17] Zhao M, Lu T, Huang Y, Yin J, Jiang T, Li M, et al (2019). Survival and Long-Term Cause-Specific Mortality Associated With Stage IA Lung Adenocarcinoma After Wedge Resection vs. Segmentectomy: A Population-Based Propensity Score Matching and Competing Risk Analysis. Front Oncol.

[18] Gu Z, Sun J, Wang L (2024). mRNA expression insights: Unraveling the relationship between COPD and lung cancer. Journal of Gene Medicine.

[19] Hopkins R J, Young R P (2019). Mevalonate Signaling, COPD and Cancer: The Statins and Beyond. Journal of Investigative Medicine: The Official Publication of the American Federation for Clinical Research.

[20] Yi X, Gao J, Wang Z (2022). The human lung microbiome: A hidden link between microbes and human health and diseases. Imeta.

[21] Gosens R, Giangreco A, Sahai E, Chambers R C (2017). Mechanistic overlap between chronic lung injury and cancer: ERS Lung Science Conference 2017 report. European Respiratory Review: An Official Journal of the European Respiratory Society.

[22] Caramori G, Ruggeri P, Mumby S, Ieni A, Lo B F, Chimankar V, Donovan C, Andò F, Nucera F, Coppolino I, Tuccari G, Hansbro P M, Adcock I M (2019). Molecular links between COPD and lung cancer: New targets for drug discovery?. Expert Opin Ther Targets.

[23] Rooney C, Sethi T (2011). The Epithelial Cell and Lung Cancer: The Link between Chronic Obstructive Pulmonary Disease and Lung Cancer. Respiration: International Review of Thoracic Diseases.

[24] Meng L, Xu R, Li J, Hu J, Xu H, Wu D, et al (2024). The silent epidemic: Exploring the link between loneliness and chronic diseases in China's elderly. BMC Geriatr.

[25] Wang Z (2013). Association between chronic obstructive pulmonary disease and lung cancer: The missing link. Chin Med J (Engl).

[26] Eapen M S, Mcalinden K D, Myers S, Lu W, Sohal S S (2019). microRNAs Are Key Regulators in Chronic Lung Disease: Exploring the Vital Link between Disease Progression and Lung Cancer. J Clin Med.

[27] Bozinovski S, Vlahos R, Anthony D, Mcqualter J, Anderson G, Irving L, Steinfort D (2016). COPD and squamous cell lung cancer: Aberrant inflammation and immunity is the common link. Br J Pharmacol.

[28] Salvato I, Ricciardi L, Nucera F, Nigro A, Dal Col J, Monaco F, Caramori G, Stellato C (2023). RNA-Binding Proteins as a Molecular Link between COPD and Lung Cancer. COPD.

[29] Hou W, Hu S, Li C, Ma H, Wang Q, Meng G, et al (2019). Cigarette Smoke Induced Lung Barrier Dysfunction, EMT, and Tissue Remodeling: A Possible Link between COPD and Lung Cancer. Biomed Res Int.

[30] Rami-Porta R, Bolejack V, Giroux D J, Chansky K, Crowley J, Asamura H, Goldstraw P (2014). The IASLC Lung Cancer Staging Project: The New Database to Inform the Eighth Edition of the TNM Classification of Lung Cancer. J Thorac Oncol.

